# Effect of Ultrasound on the Compaction of Ibuprofen/Isomalt Systems

**DOI:** 10.3390/pharmaceutics1010003

**Published:** 2009-10-09

**Authors:** Adamo Fini, Cristina Cavallari, Francesca Ospitali

**Affiliations:** 1 Department SMETEC, University of Bologna, Via San Donato, 15, 40127 Bologna, Italy; 2 Department of Pharmaceutical Sciences, University of Bologna, Via Belmeloro, 6, 40126 Bologna, Italy; 3 Department of Physical and Inorganic Chemistry, University of Bologna, Viale Risorgimento 4, 40136 Bologna, Italy

**Keywords:** ibuprofen, isomalt, traditional and ultrasound compaction, thermal analysis, SEM, Raman and FT-IR spectra

## Abstract

Six mixtures, containing 10, 20 and 30% w/w ibuprofen and isomalt, were compacted by a traditional or ultrasound-assisted machine and analysed by means of thermal (DSC and TGA) and micro-spectrometry (Raman and FT-IR) techniques. Ultrasound discharge causes melting of ibuprofen powder, transforming into a paste that could not assume the shape of a tablet; when in mixture with isomalt, thermal events, occurring during ultrasound compaction, change the appearance of the particles formed by milling the tablets obtained this way and SEM photos reveal a dramatic reduction of the particle size and changes due to a possible ibuprofen re-crystallization. Raman and FT-IR spectra of small portions of the surface and of the bulk, using characteristic peaks for identification, reveal that in ultrasound-compacted tablets ibuprofen partially disappears from the top face of the tablet.

## Introduction

Ultrasound has been used for many years to assist the compression of metallic, ceramic or plastic materials, but represents still a novel method in the field of powder compression in the pharmaceutical Industry [1]. The application of this technique to pharmaceutical technology would add a new dimension to conventional compression techniques and allow for the development of new products with new properties: a machine developed for pharmaceutical purposes was described in recent papers and some preliminary results were described in the preparation of matrixes for sustained release of theophylline [2,3]. The lack of systematic knowledge of physics underlying the ultrasound-assisted compaction of pharmaceutical powders prevented optimizing the processing steps for industrial pharmacy utilization. Many groups are involved since many years in the application of ultrasound in compaction with the aim to design a controlled release of a variety of active agent from systems thus prepared, taking the advantage from the physical changes occurring during the ultrasound compaction [4,^5^,^6^,^7^,^8^,^9^,^10^,^11^,^12^] and the possibility to obtain a direct formation of tablets even with powders of poor compactability. It is well known that pharmaceutical powders experience plastic or elastic deformation as well as fragmentation into smaller particles under the influence of an applied pressure and the formation of a tablet, starting from powders, depends on the physical behaviour of the particles. Ibuprofen and paracetamol, for instance, undergo fragmentation and elastic deformation and these processes do not allow the formation of a tablet by direct compression, even though the presence of moisture promote plastic flow under applied pressure [13]. Levina *et al*. [14,15,16] found on the contrary that coherent ibuprofen or paracetamol tablets could be prepared by ultrasound assisted compaction at pressure as low as 20-30 MPa. The explanation provided for enhanced compactability was that this type of compression process can cause a localized temperature rise within the compact originating material sintering as a consequence of progressive transformations of the pores within the tablet; and even partial and localized melting and subsequent fusion of particle surface. As a consequence low melting drugs and/or excipients can behave as a binder, increasing interparticulate bonding and behave suitably for direct compaction under ultrasound. This aspect of ultrasound effect during compaction does not appear to be explored and discussed in all its consequences and the melting of an active agent was presented as a hypothesis. In present paper we describe further aspects of the ultrasound compaction in systems containing ibuprofen and isomalt as excipient, employing techniques scarcely used for problems in pharmaceutical technology. 

Ibuprofen is a white powder with a low melting point of 74-77°C, whose behaviour under ultrasound compaction was reported and discussed [14]. Since it was also measured [8] that under ultrasound compaction local temperature of the tablet can rise up to 50-60°C, the presence inside the compacting powder of a low melting compound can favour to evidence this phenomenon. To this purpose the drug was associated to isomalt that is an artificial sugar substituted, stable to heating and relatively high melting excipient (145-150°C). Isomalt was chosen for its excellent compactability that in turn could improve the direct compaction of ibuprofen and the difference in melting point could allow evidencing only thermal changes concerning ibuprofen. Other physical and chemical characteristics of isomalt demonstrated suitable to support present research and will be shown in the Discussion below.

Raman and FT-IR micro-spectroscopy and thermal analysis, both on compact and on particulate obtained by milling, were used for present research, as well as SEM observations. The spectroscopic techniques were successfully applied to study the systems PVP/indomethacin in the form of tablets to evaluate possible physical effects of ultrasound discharge on the system components [17] and to highlight the structure of inclusion complexes of progesterone with hydroxypropyl-β-cyclodextrin [18,19]. Micro-spectroscopy provides structural identification and quantitative analysis, is non-invasive and non-destructive and particularly adequate to study intra- and intermolecular interactions and allows a comparative investigation of drug performance in the solid state when mixed with the required excipients in the formulation of a tablet [20,21].

## Experimental Part

### Materials

Ibuprofen and isomalt were commercial samples of pharmaceutical grade (ibuprofen: Fluka, Buchs, Switzerland; isomalt DC: Süβungsmittel, Mannheim, Germany) and used as received. For the present purpose commercial isomalt that contains two water molecules of crystallization was dehydrated in oven (two days at 70°C) and only this form was used throughout the paper to prepare physical mixtures and tablets.

### Methods

*Traditional Compaction -* The components (each of the size fraction 75-150 mμ) of the mixtures were added in the form of powders into a mixer and stirred and blended for 15 min at 60 rpm in a tumbling apparatus (Turbula T2c, Bachofen, Basel, Switzerland). A typical formulation contained: ibuprofen 10%, isomalt 87% and lubricant (talc + magnesium stearate) 2% w/w. One gram of each formulation was directly compacted using a single punch tabletting machine (Korsch type EKO, Berlin, Germany) to tablets (11 mm diameter). The standard applied compression force was 50 kN; for comparison, some tablets were prepared using lower force (10–40 kN).

*Ultrasound-Assisted Compaction -* For most experiments one gram of the physical mixture was separately weighed and manually loaded into the die. The physical mixtures were compacted by a ultrasound-assisted compaction machine, operating at 20 MHz and producing tablets of 2.5 cm diameter. Tabletting was carried out on the ultrasound compaction rig, providing compaction pressures of up to 6 bars together with high-power ultrasound vibration. Different tablet batches were obtained by setting the ultrasound energy between 600 J and 350 J. Tablets thus obtained were milled and sieved, using powders of 75–150 μm dimensional size for further examinations. Ultrasound parameters (power output and ultrasound time) were set up before compression by controls available on the rig. Amplitude was monitored by means of the direct reading amplitude meter and manually recorded. Before each compression, the face of the horn and die wall was cleaned with acetone, obtaining flat-faced tablet.

*Scanning Electron Microscopy -* Surface characteristics of tablets and tablet fractions were assessed using a JSM-T200 scanning electron microscope (SEM) (JEOL, Japan). Samples of whole tablets or tablet fractions were first mounted onto aluminium studs using silver-deg adhesive. The specimens were positioned so that a punch contact surface or a fractured edge could be seen. The studs were then placed in the coating chamber. The chamber was evacuated, refilled with inert gas, and the samples were coated with gold emitted sputtered at 1.2 kV. 

*Fractal Analysis -* The fractal analysis was carried out using SEM interfaced to an IBM PC. Fractal dimension of the particle contour can be obtained by SEM by means of suitable programs: it ranges from 1, for the regular contour of an Euclidean geometrical object, to 2 for a very irregular particle perimeter. With an easy extension, provided that a statistically significant number of determinations are carried out, the fractal dimension of a particle contour can be used to define the roughness and irregularity of its surface. 

*Differential scanning calorimetry analysis -* Thermal characteristics of the pure materials, the physical mixtures, the final tablets, both prepared by traditional and ultrasound-assisted compaction, were determined by an automatic thermal analyzer system (Mettler 821^e^). The data processing system (Mettler 821^e^/500/847 thermo-cryostat) was connected to the thermal analyzer. Sealed and holed aluminum pans were used for the analysis for all the samples. Temperature calibrations were made using indium as standard. The thermograms were run at a scanning rate of 10°C/min, from 30 to 200°C and the results are the mean of triplicate analysis.

*Thermogravimetric analysis (TGA) -* Thermogravimetric analysis was performed with a Mettler Toledo automatic thermal analyzer system TGA/SDTA851^e^/SF/1100). Open alumina crucibles were used for analysis in the temperature range 30-300°C at 10°C/min scanning rate under nitrogen stream.

*X-ray Diffractometric Analysis (XRD) -* To perform X-ray diffractometric analysis a Philips PW 3719 diffractometer was used, controlled by a computer. Experimental conditions were as follows: Cu Kα radiation (λ = 1.78896 Å); 40 kV and 30 mA. Scanning interval: 5-50° 2θ; Time per step: 1s; Graphite monochromator on the diffracted beam. 

*Micro-Raman spectroscopy* – Spectra were recorded by means of a Renishaw Raman Invia interfaced to a microscope Leica DMLM (maximum spatial resolution: 1 μm^2^). Experimental details: sources: Laser Ar^+^ (514.5 nm), Diode Laser (780.0 nm); monochromators: 1800 and 1200 lines/mm; detector: CCD (*Charge-Coupled Device*) cooled at 203K; spectral resolution: 2 cm^-1^; power on the sample 0.3 – 3.0 mW; accumulation time: 30 s; scansion number = 1. It was chosen to employ a diode laser with respect to an Ar^+^ laser for higher intensity of the peaks.

*Micro-Raman mapping -* Spectra were recorded by means of a Horiba Jobin-Yvon T64000 triple monochromator spectrometer interfaced to a Olympus BX40 confocal microscope (maximum spatial resolution: 0.9 mm) and equipped with a motorized xy-stage. Experimental details: source: laser Kr^+^ (647.1 nm); monochromators 1800 lines/mm; detector: CCD (charge-coupled device) cooled at 140 K; spectral resolution 0.3 cm^-1^; power on the sample: 0.5-4 mW; accumulation time 60 s; scansion number: 2; points for each mappature 8x10.

*Micro-FT-IR spectroscopy–* ATR spectra were recorded by a Nicolet FT-IR Nexus 470 connected to a Nicolet Continuum microscope: Experimental details: source globar (SiC candle); beam splitter m-IR: KBr; detector: MCT (CdTe, doped by Hg); spectral window: 4000-650 cm^-1^; lateral resolution: 7-80μm; spectral resolution: 4 cm^-1^. 

## Results and Discussion

Ibuprofen exists as a colourless crystalline solid with no reported polymorphs: it is a weakly acidic, implying low aqueous solubility in acidic pH media (water solubility: 0.05 mg/mL at 25°C) [22]. For the present research it was formulated with isomalt, an artificial sugar substitute, formed by an equimolecular mixture of 1-O-alpha -D- glucopyranosyl-D-mannitol di-hydrate (GPM) and 6-O-alpha -D- glucopyranosyl-D-sorbitol (GPS). This excipient is stable to heating and melts at a temperature between 145 and 150°C. Tablets of the ibuprofen/isomalt systems were prepared under traditional as well as ultrasound-assisted compaction to explore the differences obtained with these processes. Tablets were examined as particulate after crushing and *in toto* by a variety of analytical techniques, such DSC, TGA, micro-FT-IR and Raman, and SEM to evidence the effect of ultrasound compaction.

**Figure 1:**
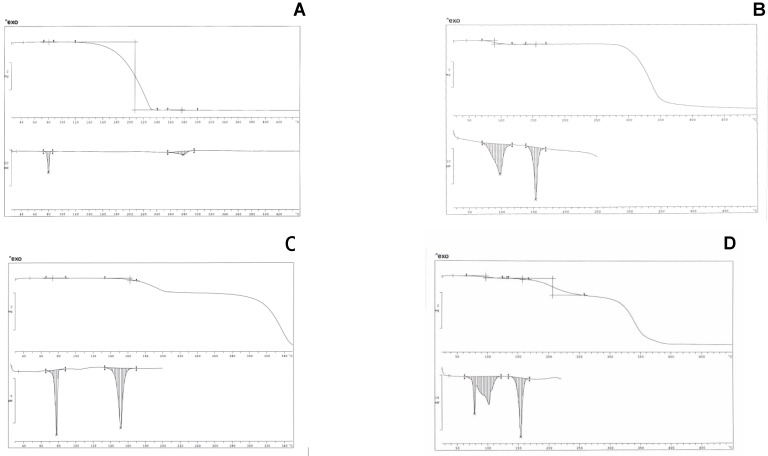
TGA profiles (above) and DSC thermograms (below). A – ibuprofen; B – hydrate isomalt; C – ibuprofen (10% w/w) and de-hydrated isomalt mixture; D - ibuprofen (10% w/w) and hydrate isomalt mixture.

*Thermal analysis* –A preliminary thermal analysis of the ibuprofen/hydrate isomalt revealed that both endotherms of ibuprofen melting and isomalt dehydration partially overlap, thus preventing reliable measurements of thermal parameters of the systems. As a consequence isomalt was previously dehydrated, by heating in oven, and only the results for the systems containing de-hydrated isomalt were reported. Figure 1 A-D show TGA and DSC thermogram profiles for the pure components and for two mixtures containing ibuprofen with hydrate and de-hydrated isomalt. 

Ibuprofen is a pure crystalline material: the DSC trace of pure ibuprofen exhibits a symmetric melting endotherm at 78°C (ΔH = 125±5 Jg^-1^). 

TGA does not indicate loss of weight in the melting temperature range, suggesting that the drug is not hydrated or solvated. Above the melting temperature, starting from 110°C up to about 230°C, a massive loss of weight (>98%) can be observed in TGA profile, probably related to its evaporation, not associated however to any (endo/exo) thermal peak in thermogram in the same temperature range; only an asymmetric endothermic peak (ΔH = 94±10 Jg^-1^) is present in the thermogram in the range 260-80°C (Figure 1 A). 

Isomalt actually is an equimolar mixture of two stereoisomers (glucopyranosyl mannitol – bi-hydrate: GPM.2H2O and glucopyranosyl sorbitol, GPS) [23,24]. Thermogram of pure isomalt shows two endotherms (Figure 1 B). The first one at 97.5°C (peak temperature), associated to a loss of weight (4.93% in the temperature range 70-130°C, that is one water molecule for one isomalt molecule) is broad and asymmetric and can be attributed to GPM dehydration: the high ΔH = 160±10 Jg^-1^ associated to dehydration suggests the existence of strong interactions between the water crystallization molecules and the high number of hydroxy groups present in the excipient molecules: this endotherm decreases its area under the peak after heating in oven at 70°C and shifts to lower temperature its peak, disappearing after a two-day heating at this temperature. The second endotherm, more symmetric and narrow, is related to the melting of the sugar drug (153°C, ΔH = 127±10 Jg^-1^). This endotherm decreases its area under the peak of about 10% when measured for the hydrate sample, suggesting that dehydration leaves the solid mass partially amorphous. Dehydration at slow rate (heating in oven at 70°C) allows complete recover of crystallinity. On heating, isomalt is stable up to about 250°C; after dehydration isomalt tends to absorb humidity from air, in the absence of any protection.

When the ibuprofen/isomalt physical mixtures at different compositions are considered, it is possible to appreciate in the thermogram (30-450°C) the presence of only two endotherms, whose identification was attributed by comparison with thermograms of pure compounds. The first endotherm (centred at 78°C) has an area under the peak increasing at increasing ibuprofen concentration that can be attributed to the melting of the drug; the second one is related to melting (centred at 153°C) of de-hydrated isomalt. A third endotherm, irregular and broad (~280°C), related to the evaporation of ibuprofen, is usually present in thermograms: since for further tests temperature scanning was limited to the range 30-200°C, this last endotherm was not shown (Figure 1 C). Figure 1 D shows the interference between the endotherm of melting of ibuprofen and that of the dehydration of the hydrate isomalt: this fact suggested the previous dehydration in oven of the excipient. Similar thermograms were obtained for the same samples after traditional compaction into tablets. Calculation carried out on both systems let us to draw the conclusion that thermal parameters of ibuprofen and isomalt do not change when they are in a physical mixture or in traditionally compacted tablets, suggesting the absence of any interaction, maintaining both compounds the starting crystallinity.

On the contrary, in thermograms of the samples containing de-hydrated isomalt and obtained under ultrasound compaction, the area under the peak of the ibuprofen melting endotherm was markedly reduced. This fact can be attributed to thermal events associated to ultrasound compaction. Ibuprofen demonstrated a particular behaviour during thermal treatments. During a thermal cycle, a sample of ibuprofen, previously melted, does not recover its crystallinity on cooling and the sample soon after this treatment did not show any melting peak. Powders of pure ibuprofen compacted under ultrasound transformed into a soft paste that solidified and crystallized only after several days. It can be hypothesized that these phenomena could occur also when ibuprofen is in the presence of excipients.

As a consequence the decrease of the area under the peak of ibuprofen melting endotherm, when in mixture with de-hydrated isomalt and compacted under ultrasound, can be attributed to its amorphization following the melting under ultrasound discharge. Moreover thermogravimetric analysis of tablets allowed to ensure that ibuprofen does not decompose under ultrasound: in each case the loss of weight associated to the evaporation of ibuprofen in the thermogravimetric profiles agreed better than 98% with the nominal value of the concentration at all the compositions examined. 

An estimation of crystalline ibuprofen content was obtained by DSC data, comparing the area under the peak of ibuprofen melting endotherm measured in the presence of both types of compaction and for the untreated drug in the absence of the excipient and calculated by:

Percent crystallinity = [ΔH_t_/(ΔH_p_. C)]. 100

where *ΔH*_t_ and *ΔH*_p_ are enthalpies of fusion of ibuprofen when in tablets or when pure, respectively, and *C* is the experimental weight fraction of the drug in the mixture. The equation assumes that the pure drug was 100% crystalline. Since the literature does not describe different polymorphic forms for ibuprofen, reduced area under the peak can be originated from decreasing drug crystallinity and not from altered polymorphic forms of ibuprofen. Table 1 shows the results of DSC analysis of the tablets compacted under ultrasound and reveals that calculated crystallinity of ibuprofen is reduced (to an extent ranging from 40/50%, for 10% ibuprofen content, to 25/30% for higher concentration) with respect to the starting value and appears to increase as ibuprofen content increases inside the tablet. 

This can be due to the fact that at low concentration ibuprofen is dispersed inside a large mass of the excipient, while at higher concentration it is easier for ibuprofen to re-build its crystalline lattice after the melting under ultrasound discharge. However, since the DSC approach essentially shows this parameter only at the temperature of determination (i.e. at the melting point of ibuprofen), it cannot be excluded an (at least partial) recover of crystallinity during temperature scanning up to 78°C.

**Table 1:** Composition of the ibuprofen/(de-hydrated) isomalt systems compacted under ultrasound and analyzed by DSC. Pure Ibuprofen: ΔH = 125.7 Jg^-1^.

Sample*	Energy released/Pressure (J/bar)	ΔH_melting_ (Jg^-1^)	% crystallinity
			
10% Ibuprofen			
1	500/5	7.0	55
2	550/5	4.6	37
3	600/5	5.1	40
			
4	500/6	7.0	55
5	550/6	8.1	64
6	600/6	6.7	53
			
20% Ibuprofen			
1	400/5	17.2	68
2	450/5	16.6	66
3	500/5	19.0	76
			
4	400/6	19.5	78
5	450/6	20.0	79
6	500/6	18.2	72
			
30% Iuprofen			
1	300/5	31.0	82
2	360/5	29.6	78
3	400/5	28.2	75
			
4	250/6	27.6	73
5	285/6	30.0	79
6	329/6	30.2	80

*The samples contained 1% talc and 1% magnesium stearate.

*SEM images* – Qualitative inspection of the tablets by means of electron microscopy was carried to evaluate the effects of ultrasound on the microstructure of the tablet components after ultrasound-assisted compaction. These results were obtained using the particles obtained by milling tablets, prepared under ultrasound, and the whole tablet.

**Figure 2:**
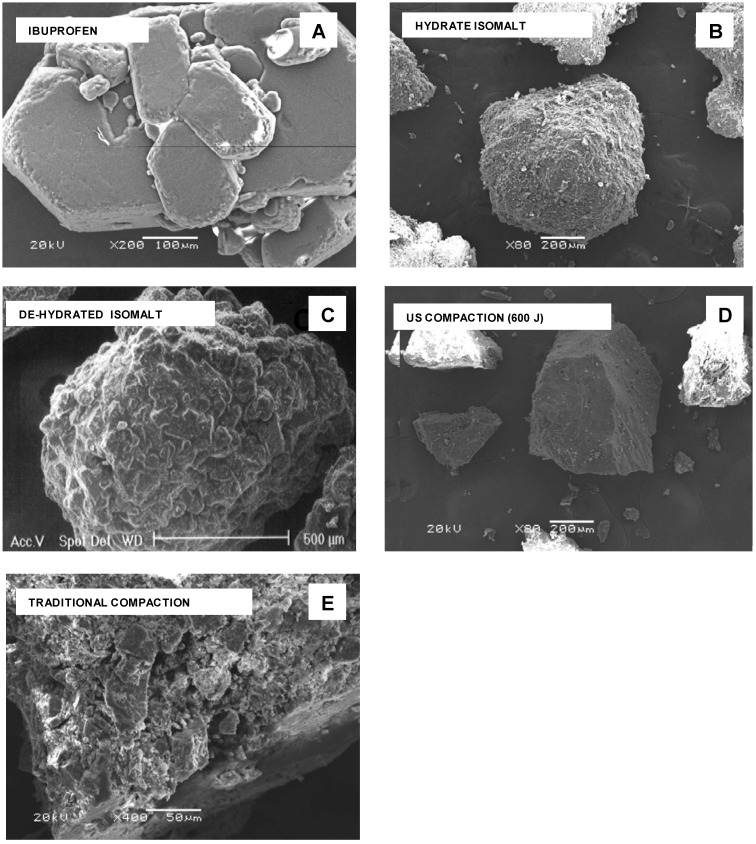
SEM photos. A – ibuprofen; B - hydrate isomalt; C – de-hydrated isomalt; D – tablet fragment from ultrasound-assisted compaction; E - tablet fragments from traditional compaction.

At SEM examination, particles of pure compounds appear with morphology very different from each other (Figure 2 A-D). Ibuprofen particles have a crystalline habit with agglomerates formed by hexagonal plates stacked together (Figure 2 A), while isomalt shows agglomerated spherical particles with rounded margins and irregular and rough surface (Figure 2 B): this morphology was practically retained after dehydration (Figure 2 C). After milling of a tablet compacted under ultrasound, particles show sharp edges, compact texture and fairly smooth surface (Figure 2 D): in fragments of a tablet traditionally compacted, single component regions appear assembled together with deformed edges and also reduced size, following the pressure of the die (Figure 2 E).

**Figure 3:**
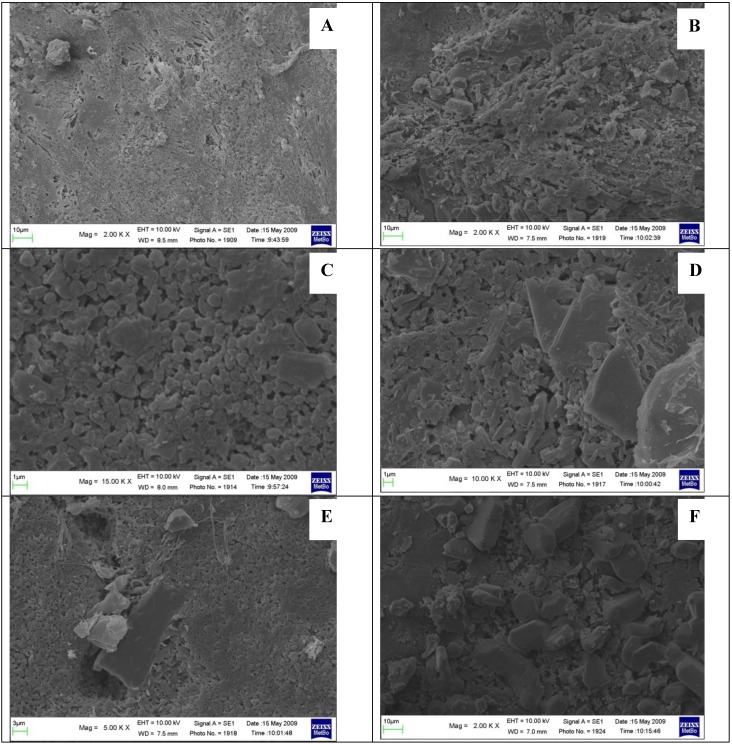
SEM photos. A – top face (2kx); B – inferior face (2kx); C – top face (15kx); D – re-crystallization (inferior face: 10kx); E – re-crystallization (inferior face: 5kx); F – re-crystallization (inferior face: 2kx).

As a consequence the fractal dimension of the particle contour was found lower for powder obtained under ultrasound compacted tablets (1.10) than for conventionally compacted ones (1.25). This fact agrees with what reported [16] that tablets prepared under ultrasound have smoother surface in comparison to the surface of the conventionally prepared tablets. Fusion of particles at surface asperities, under ultrasound discharge, that increases interparticulate bonding reducing void spaces is thought responsible of these new characteristics, acquired by the particulate under ultrasound. Even though rises in bulk temperature up to 36°C were recorded during ultrasound compaction of ibuprofen [16], this does not eliminate the possibility of local temperature rises at the contact and friction points of the powder particles above the melting temperature of ibuprofen (78°C). As a result the melting, the area of contact between the powder particles increases; the molten material solidifies to form solid bridges and acts as a binder inside the tablet: this produces smoothing of the contact surface at the interfaces tablet/die, but also at the fracture surface when tablets are crushed. Ultrasound compaction operates also in reducing particle size inside the tablet: the surface of a tablet appears heterogeneous only at very high magnification (particle size ≈ 1 μm); otherwise it appears continuous and compact (Figure 3 A).: At accurate SEM examination the two faces of the tablet appear different, being the surface of the inferior face more irregular than the superior one (Figure 3 B): this was expected due to mode of ultrasound-assisted compaction, where ultrasound operate only on the top of the tablet. In the inferior face it is frequent to encounter presence of plates of almost regular shape (visible at high magnification). The shape of these micro-plates recalls that of ibuprofen crystals, shown in Figure 2 A that could suggest an asymmetric distribution of the drug. To test this hypothesis we employed micro-spectroscopy Raman and FT-IR (see below).

It can be hypothesized that these plates have been formed after some event occurred following the ultrasound compaction, since their shape allows identifying them as clearly different from the circumferencing areas and can be attributed to ibuprofen crystals formed after the melting under ultrasound discharge. Moreover these areas are less frequent in the top surface, where the background appears more regular (Figure 3 A and Figure 3 B), differently from the opposite surface, more irregular (Figure 3 C) and where the formation of crystals (Figure 3 D), whose shape closely recalls that of ibuprofen hexagonal plates shown in the Figure 2 A. Figure 3 E and Figure 3 F again show the differences between the two surfaces when the electron beam modifies the surfaces by some thermal effect.

*Raman and IR spectra* – Tablets obtained by means of the two different methods were examined by Raman and IR micro-spectroscopy: since these techniques allow a direct observation of the sample, without any special preparation procedures that could alter their characteristics [25]. 

**Figure 4:**
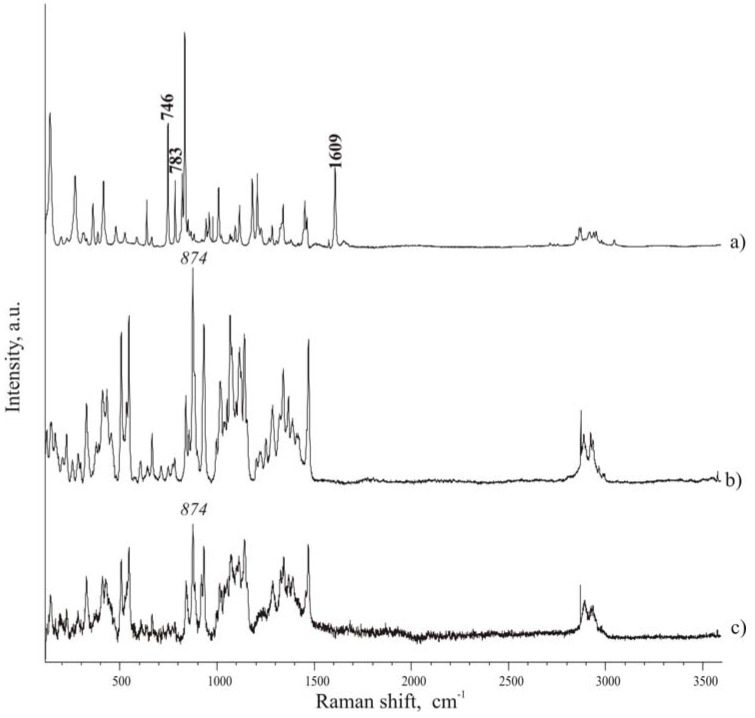
Micro-Raman spectra. A – ibuprofen; B – hydrate isomalt; C – de-hydrated isomalt.

Raman spectra of pure components were first registered as a reference; then spectra were also collected for the tablets the in the spectral range 100-3500 cm^-1^. Spectra of compounds (Figure 4) are rich of narrow and well defined peaks, whose wave number position in the considered spectral range can be employed to identify each compound, when in mixture. Recent papers [26,27] report a precise attribution of the bands in the Raman spectrum of ibuprofen: in the present paper however we are not interested in the intrinsic assignment of the bands, rather in the use of distinctive bands to evidence the presence of ibuprofen inside the formulation. By comparison of the spectra of the two components (Figure 4 A and Figure 4 C), it was possible to consider the following peaks to check the presence of ibuprofen in the mixtures: 746, 783, 1609 cm^-1^, which can be found in spectral regions where isomalt peaks do not overlap and interfere. Particularly interesting are the band at 746 cm^-1^, associated to out-of-plane deformation of C-H group of an aromatic ring, and the band at 1609 cm^-1^, attributed to the stretching of aromatic C–C bonds (in-plane ring deformation and C–C ring stretching). Since few pharmaceutical excipients contain aromatic groups, thus the spectral regions characteristic of aromatic C−C and C−H stretching usually have peaks arising only from the active substance (in addition to functional groups present in the drug and not commonly encountered in excipients). 

The same band was chosen by other authors to evidence the physical state of ibuprofen in solid dispersion with PVP (1613 cm^-1^) [28]. 

The band at 874 cm^-1^ of isomalt spectrum was chosen as reference peak for the excipient.

**Figure 5:**
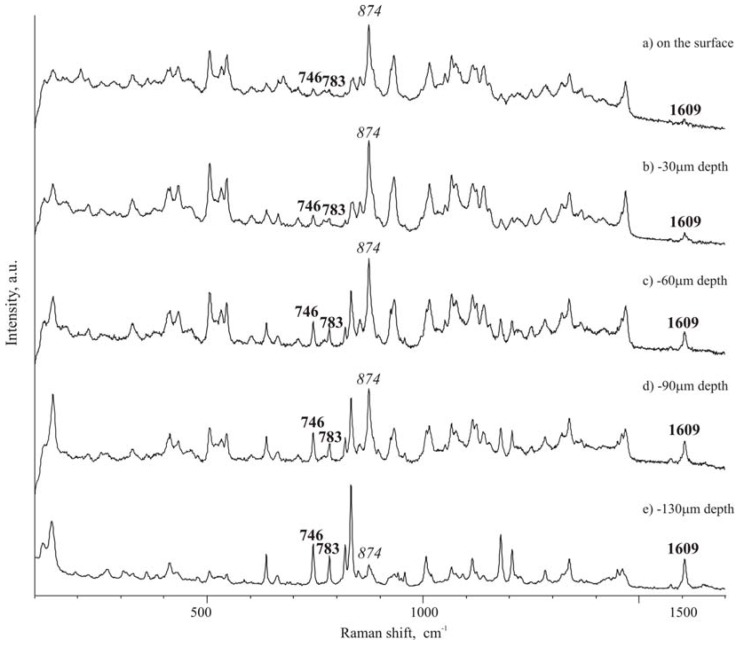
Micro-Raman spectra along the section of a tablet: A – top surface (level 0);B – -30 μm; C – -60 μm; D – -90 μm; E – -160 μm.

To evaluate possible asymmetry of ibuprofen distribution, from the top to the opposite face of a tablet, Raman spectra were carried out in different points of the cross-section of a tablet. Traditional tablets show a discrete distribution of the drug and excipient along the section of the tablet, as that observed for the surface of the tablet. On the contrary, in ultrasound-compacted tablets it was found that ibuprofen peak at 1609 cm^-1^ increases its intensity from the top surface of the tablet towards the bulk of the tablet: passing from the top surface, as level 0, where practically only isomalt could be detected by the high intensity peak at 874 cm^-1^ (Figure 5 A), to lower levels it appears that the ibuprofen peak at 1609 cm^-1^ starts to be appreciable at 30 μm below the top surface and, at 130 μm level, ibuprofen peak appears dominant over that of isomalt (comparing intensity of the two reference peaks) (Figure 5 B-D). This phenomenon is less accentuated when ultrasound energy of compaction was lower.

On the contrary on the surface of the opposite side it can be appreciated an increase of the ibuprofen concentration, as documented by an increase of the frequency, with which the reference peak of ibuprofen is encountered.

Spectra of the Figure 5 is reported as an example of those registered along a line starting from the level 0 (top surface) toward the inner regions of the tablet. Other tests gave statistically similar results indicating a progressive increase of the intensity of the ibuprofen reference peak.

The same results were obtained using IR spectra. In this case the band of the carboxyl at 1710 cm^-1^ (C=O stretching) was chosen as reference peak in the IR spectrum of ibuprofen, both since it is the most intense peak of the spectrum and since isomalt does not present any peak in this spectral region. This band was absent in the Raman spectrum [29]. 

When the tablets surface was examined, a similar behaviour occurred as that previously noticed with Raman microscopy. On the traditional tablet it is possible to observe (not shown here) distinctly separated both pure compounds. In FT-IR spectra, collected on a number of portions of the ultrasound-compacted tablet top surface, the presence of ibuprofen was attested through its reference band at 1710 cm^-1^ only in very few points of the tablet surface. While FT-IR more frequently could detect the presence of isomalt. In tablets compacted under higher ultrasound energy, reference peak of ibuprofen, when detected, was found shifted to 1722 cm^-1^. This could indicate a modified carboxyl environment related to the disruption of the dimeric entities dominant in the condensed phase. 

**Figure 6:**
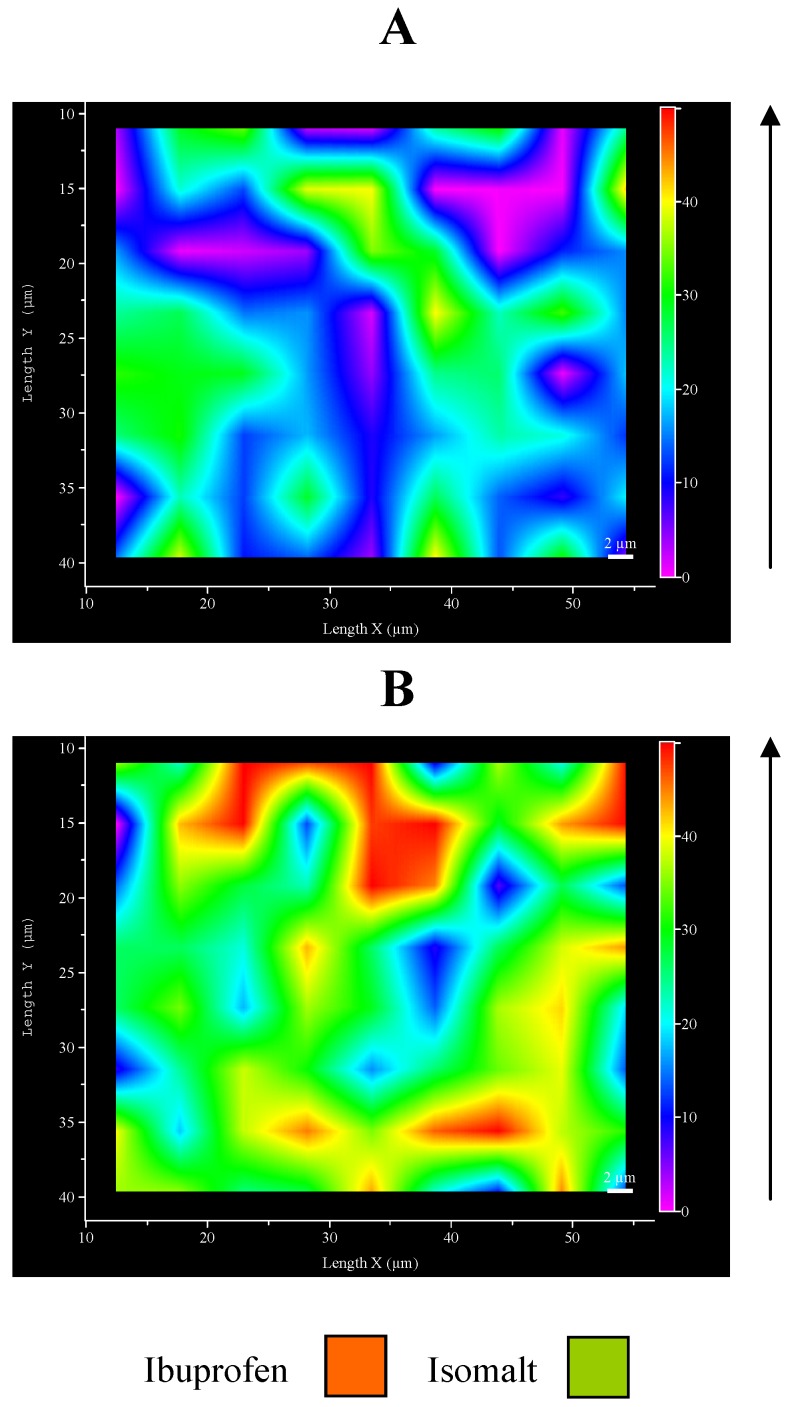
Micro-Raman maps of intensity at 1609 cm^-1^: A – top surface; B – inferior face.

This unexpected aspect of ultrasound compaction suggested preparing a map of the ibuprofen distribution on the tablet surfaces. Figure 6 represents an example of Raman map of surface portions: the different distribution of drug and excipient is evidenced by different colours: examination of different points of the surfaces produced comparable maps. This result allows to confirm previous hypothesis, since it can be clearly appreciated the major presence of ibuprofen in the inferior face with respect to the top one.

These preliminary results suggest an asymmetric distribution of ibuprofen inside the tablet, observed as a consequence of ultrasound compaction, that starts from the upper surface in direct contact with sonotrode down to internal layers situated immediately below, causing a partial disappearance of ibuprofen from the top surface of the tablet and its concentration on the opposite face of the tablet. This fact can be explained, likewise the amorphization discussed before, with the melting of the drug under ultrasound: once molten, the liquid drug tends to flow downwards through the isomalt particle and is removed from the top surface, in this assisted also by the intense movement of the sonotrode that vibrates at ultrasound frequency. Additionally it must be also outlined that ibuprofen and isomalt possess very different solubility parameters [30] that prevent their mutual solubility and thus the possibility of molten ibuprofen to dissolve isomalt and slowing down or blocking the flow: this can be a key to overcome this unexpected aspect of ultrasound compaction. The use of excipients interacting somehow with the drug could prevent this flow, when a low melting drug is used under ultrasound compaction.

Further researches will be related to observe asymmetric distribution as a function of the parameters of the ultrasound compaction (energy, pressure, frequency) also to evaluate the possibility of ibuprofen loss by evaporation (that however appears to occur at higher temperature than that developed during ultrasound compaction); and also the nature of the drug will be evaluated to determine a critical melting point of the drug as well as to select new drug/excipient pairs suitable to make the drug indifferent to this aspect of the ultrasound compaction.
